# Establishing and developing a paediatric psychodermatology service and our experience of a new paediatric psychodermatology clinic during the Covid 19 pandemic

**DOI:** 10.1002/ski2.151

**Published:** 2022-08-08

**Authors:** Alison V. Sears, Rukshana Ali, Jane O’Connor, Susannah Baron

**Affiliations:** ^1^ St John's Institute of Dermatology Guy's and St Thomas' NHS Foundation Trust London UK

## Abstract

Children and young people (CYP) with skin and hair conditions are at an increased risk of mental health problems and vice versa. Current child and adolescent mental health services are already stretched and in our experience, this unique combination of symptoms and signs requires a multi‐disciplinary approach. We report our experience of establishing a paediatric psychodermatology clinic where, at each appointment, CYP are seen by a consultant dermatologist and a clinical psychologist initially jointly and then individually to ensure all viewpoints are heard and a collaborative treatment plan can be agreed. The clinic was established one month prior to the national lockdown during the COVID‐19 pandemic and the face‐to‐face model was converted to a virtual format. CYP are now seen either face to face or virtually according to CYP/parent/carer preference and this hybrid model increases accessibility and has reduced DNA rates. Referrals were received from primary, secondary and tertiary care settings. Thirty –six new patients were seen and followed‐up over a 2 year period, age range 3–17 years old. The majority of patients presented with compulsive hair pulling (trichotillomania) and medically unexplained signs (dermatitis artefacta); other problems seen were eczema, skin picking and acne. Half of the patients required additional psychology sessions. Seventy‐six percent of patients have been discharged, almost half back to the care of their general practitioner. We use pre‐ and post‐clinic questionnaires and share these and feedback from CYP/families who have found this clinic model helpful and effective.

## Graphical abstract

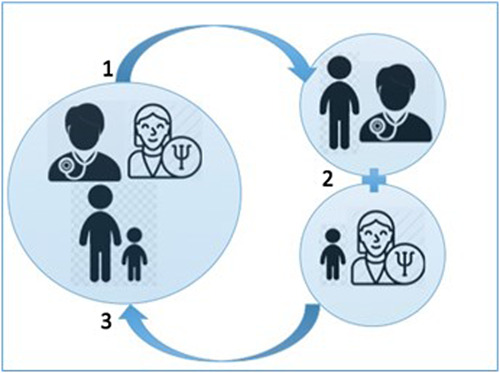

1

**What is already known about this topic?**

- Mental health problems have become more prevalent in children and young people (CYP), particularly during and after the COVID‐19 pandemic.
- Skin and hair conditions can lead to mental health issues *and* be a sign of psychological distress in CYP.
- Children and their families need health professionals with specific expertise in managing the psychological burden of skin and hair problems, which current services cannot address in a holistic way.

**What does this study add?**

- In the paediatric psychodermatology service there is an initial joint consultation between CYP, parent/carer, dermatologist and psychologist, followed by individual consultations between CYP/psychologist and parent/carer/dermatologist to ensure all voices are heard; and final joint consultation to agree collaborative action plan.
- A mixture of virtual and face‐to‐face appointments improves accessibility.
- This study demonstrates that seeing appropriate CYP within a psychodermatology clinic reduces the burden on general paediatric dermatology clinics and leads to successful resolution of symptoms and discharge.

## 2 INTRODUCTION

Skin and hair conditions are common in children and young people (CYP), and it is well known that this can cause significant psychological distress for both the CYP and their families. In addition, mental health distress and disorders have become more prevalent in CYP particularly during the Covid 19 pandemic, with almost two‐thirds reporting long term issues.^1^ In 2020 one in six children aged 5–12 years in England were identified as having a probable mental health disorder increasing from one in nine in 2017.^2^

Whilst it is well recognised that visible skin and hair conditions can greatly impact on wellbeing and cause psychological distress, it is now becoming increasingly recognised that psychological distress in children and adolescents can cause their presenting skin or hair issue. It is necessary to address both the skin/hair and psychological distress as otherwise there can be long term impact on psychological wellbeing for example, self‐esteem, confidence and mood into adulthood, which highlights the importance of early intervention^3^

This complex combination of presentations and co‐morbidities requires healthcare professionals with knowledge and experience in managing CYP within their unique family and social environment in a setting that feels safe and non‐judgemental to all. Unfortunately, pre‐pandemic there was already poor provision of care for CYP experiencing psychological distress associated with skin disease and presenting with skin conditions due to psychological/psychiatric/neurodevelopmental disorders. Furthermore, these CYP rarely meet the threshold for Child and Adolescent Mental Health Services (CAMHS). This lack of psychological support and provision was highlighted in the Mental Health and Skin Disease All Party Parliamentary Group (APPG) report in 2020.^4^ Of the 27 respondents under 18 years old, 100% indicated that their skin condition affected their psychological wellbeing and 85% had low self‐esteem, particularly in relation to engaging with peers at school. Post pandemic CAMHS are struggling to manage increasing referral numbers and severity of mental health needs, resulting in greatly increased waiting times. Moreover, children and their families need health professionals with specific expertise in managing the unique presentations and psychological burden of skin and hair problems, which CAMHS cannot address in a holistic way.

### 2.1 Establishing a new paediatric psychodermatology service

Psychology has been embedded within the adult dermatology service at St John's Institute of Dermatology for many years, however within the paediatric dermatology service CYP were referred and seen by the general paediatric psychology team within the Evelina Children's Hospital. As the general paediatric psychologists were struggling to manage increasing referrals from paediatric dermatology, a business case for a dedicated paediatric dermatology clinical psychologist was written. In 2016 there was no funding available within the Trust so we successfully applied for a 1 year pump‐prime grant (from Pharma) and appointed a band 8A clinical psychologist 0.5 whole time equivalent (WTE) in 2017. Following this we were able to demonstrate the value, effectiveness and (prior to block contract) the cost‐effectiveness of this post and were successful in business planning for a 0.6 WTE role. Our current paediatric clinical psychologist joined the paediatric dermatology department in October 2018.

Our model of care is concurrent clinics run by the clinical psychologist and the paediatric dermatology team within paediatric dermatology outpatients. Our clinical psychologist is available to see urgent within clinic referrals on the same day, and if a full psychology appointment is not possible she can meet the CYP and family with their dermatologist, which promotes engagement and rapport, destigmatises psychology and reduces anxiety and stress relating to future psychology appointments.

Within the paediatric tertiary referral severe inflammatory skin disease clinic we had recognised the increasing psychological morbidity of the CYP and their families. Having a psychologist embedded within this multi‐disciplinary team enables joint consultations and a one‐stop approach of arranging side by side clinical psychology appointments after the clinical appointment if more complex psychological intervention is needed.

However, we recognised the unmet need and further developed our service to offer care for CYP referred to dermatology with skin or hair problems which were predominantly psychologically driven for example, compulsive skin picking, medically unexplained skin signs (dermatitis artefacta), compulsive hair pulling (trichotillomania) as their needs could not be met effectively within a busy general clinic.

### 2.2 The complex clinic

In February 2020 a new monthly multi‐disciplinary paediatric psychodermatology service was established, accepting primary, secondary and tertiary referrals. All referrals are triaged appropriately and the clinic waiting time depends on the urgency of the referral. There are four 60 min appointment slots for new and follow‐up patients with consultant paediatric dermatologist, paediatric clinical psychologist, and a paediatric liaison psychiatrist available if needed. The clinic set‐up was aligned to that described by Aguilar‐Duran et al.^5^ for an adult psychodermatology service.

Prior to each appointment CYP and their families are asked to complete a questionnaire detailing their concerns and what they hope to gain from their appointment (Appendix [Link], [Link]). In addition they are asked to complete the validated Childrens' Dermatology Life Quality Index (CDLQI)^6^ and Paediatric Inventory of Emotional Distress (PIED),^7^ and the Strengths and Difficulties Questionnaire (SDQ)^8^ in clinc, if neurodevelopmental concerns are identified. There is a post‐clinic feedback form (Appendix [Link], [Link]) and this process is now electronic using the DrDoctor digital patient engagement platform.

**FIGURE 1:**
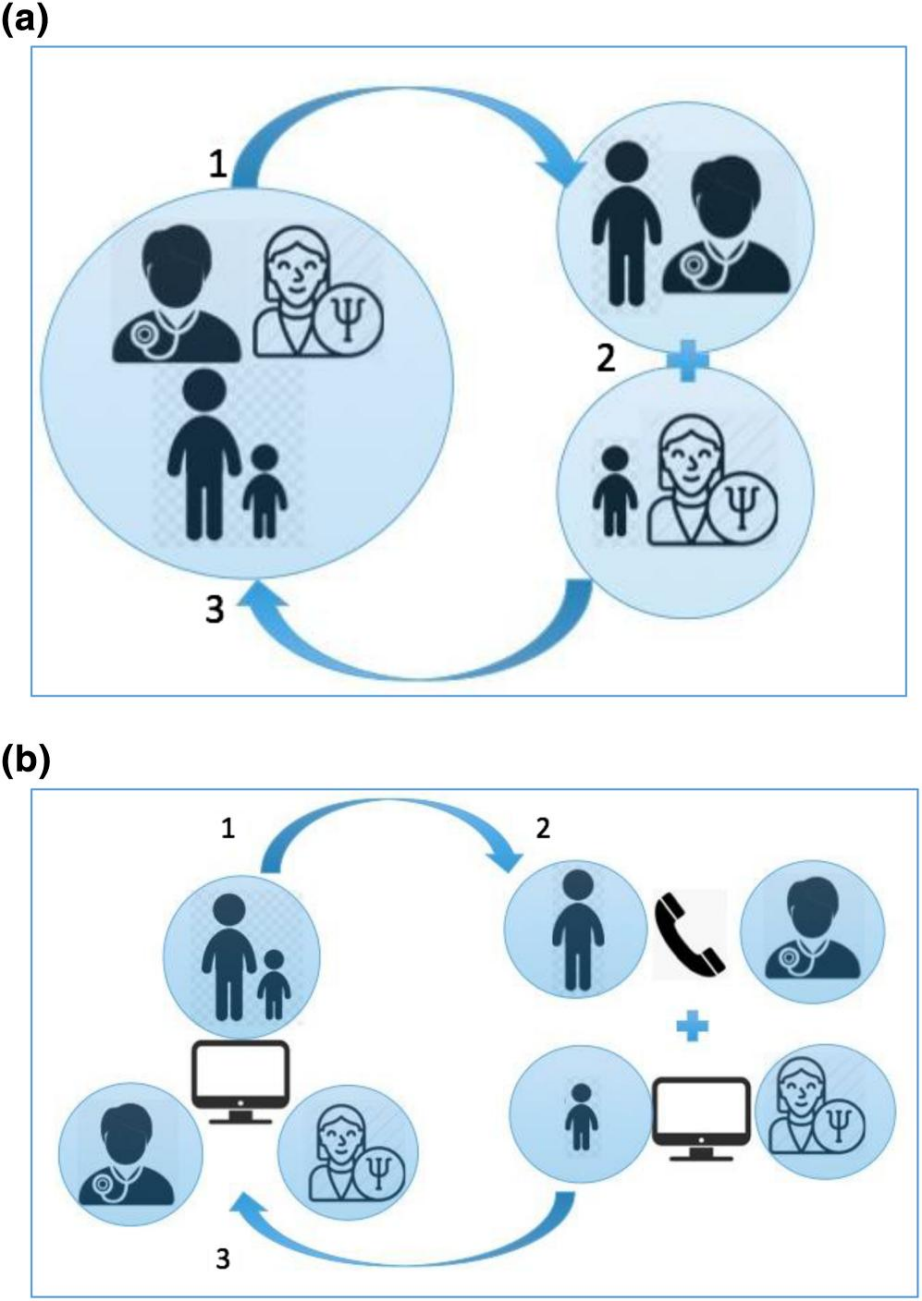
Clinic models. (a). Face‐to‐face clinic model showing (1) initial combined consultation with Children and young people (CYP), parent/carer, paediatric dermatologist and clinical psychologist; (2) separate consultations between parent/carer and paediatric dermatologist and CYP and clinical psychologist; (3) final combined consultation with CYP, parent/carer, paediatric dermatologist and clinical psychologist. (b). Virtual clinic model showing (1) initial combined video consultation with CYP, parent/carer, paediatric dermatologist and clinical psychologist; (2) continued video consultation between CYP and clinical psychologist with separate telephone consultation between parent/carer and paediatric dermatologist; (3) final combined video consultation with CYP, parent/carer, paediatric dermatologist and clinical psychologist

Prior to the Covid‐19 pandemic lockdown a face to face model of care was utilised with the CYP and their family having an initial joint consultation with the dermatologist and clinical psychologist together where the skin or hair issue is assessed together, exploring the specific impact of this on their psychological wellbeing. Then the CYP has a one to one session with the clinical psychologist who can build further rapport and explore psychological themes raised in the initial joint consultation, whilst the parent/carer continues in consultation with the dermatologist. This bi‐modal approach allows a safe and confidential space for both CYP and parent/carer and greatly adds to the therapeutic process. The final stage is for all to come together to feedback and jointly agree a psychological and clinical action plan (Figure 1a).

Unfortunately after the first clinic, the UK went into lockdown with the Covid‐19 pandemic and clinics were initially cancelled. We were determined to continue, so from April 2020 clinics were held virtually using Attend Anywhere video call management system. After an initial joint consultation, the child and psychologist remained on the video‐consultation while the parent/carer had a separate telephone consultation with the dermatologist (Figure 1b) and the joint feedback at the end was by video with all present.

Face to face consultations re‐started in July 2020 but due to the pandemic and travel distance appointments were conducted either face to face and/or virtually dependent on the CYP and families' preference.

Collection of baseline and follow‐up questionnaires prior to their electronic delivery on the digital patient engagement platform in the context of the pandemic was challenging with the combined clinic models and therefore data is incomplete. We present 2 years of descriptive data (from February 2020) reporting patient demographics, diagnoses, outcomes defined by discharges and our clinical experience.

## 3 RESULTS

**FIGURE 2:**
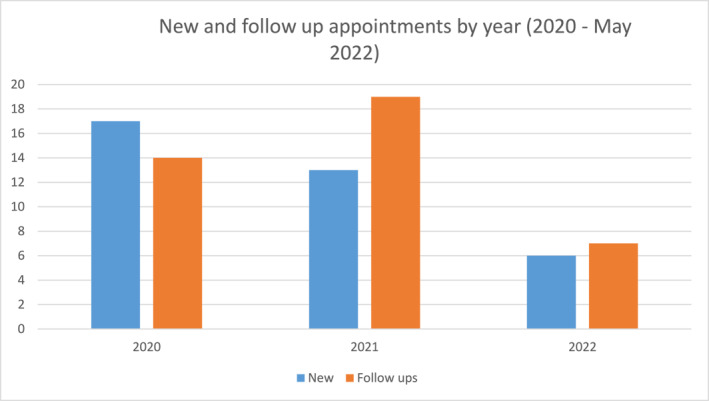
Numbers of new and follow up appointments by year (2020‐ May 2022)

**FIGURE 3:**
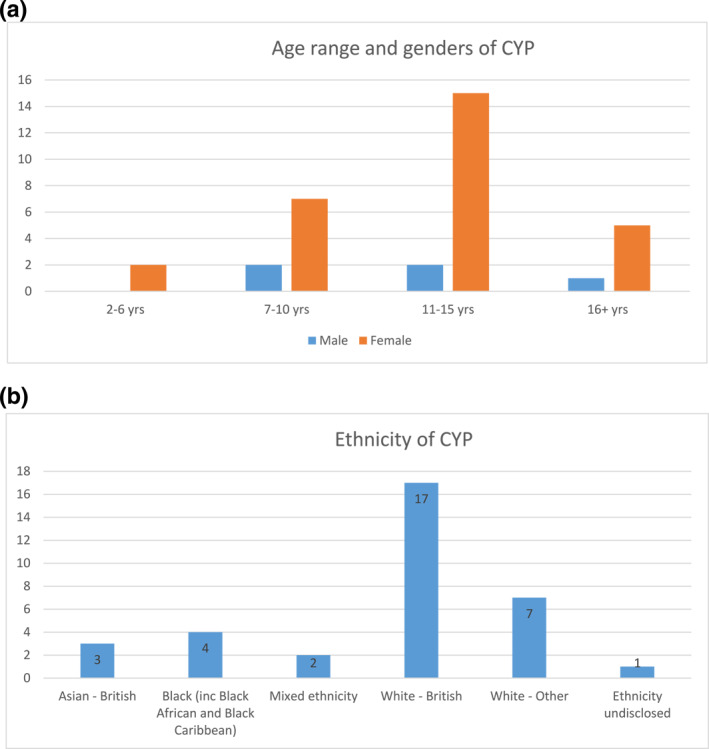
CYP Demographics. (a). Age range and gender of Children and young people (CYP). (b). Ethnicity of CYP

Since February 2020, 36 new patients were referred of which 34 attended a total of 76 appointments (36 new; 40 follow up appointments) (Figure 2). Only 2 CYP and parent/carers did not attend (DNA) their initial new appointment, (5.6% DNA rate). Children and young people median age at the point of referral was 12 years (range 2–17 years). The youngest referred CYP had turned 3 years by the time of initial assessment in the service. The most common ages of CYP at the point of referral were 12 years (*n* = 5) and 15 years (*n* = 5). The majority of CYP were female (85%; *n* = 29/34) (Figure 3a). The majority of patients were White British or White other (Figure 3b).

**FIGURE 4:**
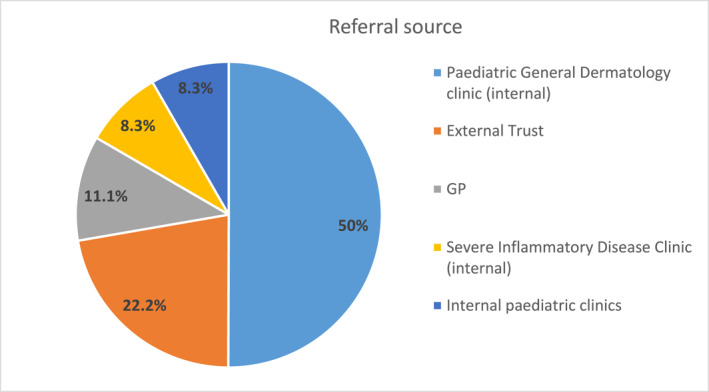
Referral source

The majority of referrals were received from our paediatric general dermatology and severe inflammatory disease clinics (50% and 8.3% respectively), followed by external referrals from other hospital trusts (22%), direct referrals from primary care (11.1%), and internal referrals from general paediatrics (8.3%) (Figure 4). Mean distance from family home address to the clinic was 25 miles (range 1.4–139 miles). The majority of patients attended one or two appointments (38% and 29% respectively) and 15% have had 4 or more appointments. One case was jointly assessed with the consultant liaison paediatric psychiatrist.

**FIGURE 5:**
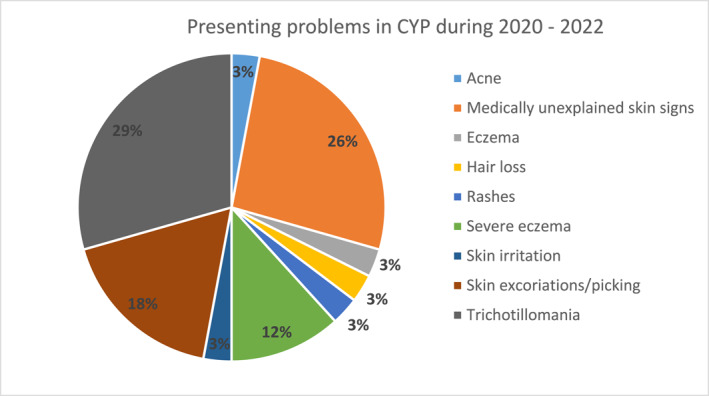
Presenting problems in Children and young people (CYP) in 2020–2022

The majority of patients presented with compulsive hair pulling (trichotillomania; 29%), closely followed by medically unexplained skin signs (Dermatitis Artefacta; 26%) (Figure 5). Of the patients presenting with compulsive hair pulling, 3 patients, also had either alopecia areata or alopecia totalis. Other presenting problems included eczema (*n* = 5), skin picking (*n* = 3) and acne (*n* = 1). Patients experiencing eczema were offered help for their itching and scratching with habit reversal and were either referred from or into a specialist inflammatory skin disease clinic.

Seventy six percent of all patients referred to the service have been discharged to date (*n* = 26). Of these, patients were seen face to face only (*n* = 9), virtually only (*n* = 3) or both (*n* = 14). Half of those discharged (50%) had additional paediatric clinical psychology appointments and the number of additional psychology appointments ranged from 1–4 sessions (separate to their reviews in the complex clinic). The majority of discharged patients were referred back to the care of their general practitioner (42.3%; *n* = 11). Other discharged patients were referred back to the specialist inflammatory skin disease clinic (15.4%; *n* = 4), general dermatology clinic (11.5%; *n* = 3), or continued with ongoing psychology (19.2%; *n* = 5); one patient self‐discharged (*n* = 1) and two were lost to follow up (*n* = 2). Two discharged patients required onward referral to CAMHS.

In 2022 to date, eight patients have completed the pre‐clinic questionnaire during a follow up visit (mean age 12 years; age range 7–17 years; female *n* = 7/8; diagnosis: skin picking (*n* = 1), medically unexplained skin signs (*n* = 4), chronic skin excoriations (*n* = 1), urticaria (*n* = 1), and alopecia (*n* = 1)). The majority (75%; *n* = 6/8) reported feeling, ‘good’ since their last visit; one CYP responded ‘very good’ and one ‘neutral’. Five reported friendships had been better or much better (*n* = 5/8; other responses: same as always (*n* = 2); worse (*n* = 1)). When asked what they would like to talk about, responses included: skin (*n* = 8/8), treatment (*n* = 5/8), hair (*n* = 1/8), school (*n* = 1/8), mood (1/8), home (*n* = 1/8), and friendships (*n* = 1/8). Expressed hopes for the appointment included: ‘to find skin isn't anything bad and know what has happened instead of worrying’ (CYP); ‘to get better, sense of normality’ (CYP and parent); and ‘get more help with skin and overthinking’ (parent). Expressed hopes for what would be different *in their life* by this time next year included: ‘to become a different person mood wise about how she looks and feels and skin healed’ (parent); ‘to have better skin and not be ashamed of how I look’ (CYP); ‘don't have to miss school and visit doctors’ (CYP); ‘not as many infections, sleep better’ (parent). The majority of the CYP (*n* = 5/8; mean age 13 years; age range 10–17) were active on social medical platforms including Snapchat (*n* = 5), Instagram (*n* = 4), TikTok (*n* = 3), Facebook (*n* = 3), and Pinterest (*n* = 1). The three CYP who did not use social media were aged 7 (*n* = 1) and 12 (*n* = 2) years.

### 3.1 Example of case

A 15 year old girl presented with oval and linear skin lesions. She was referred for a second opinion from a hospital where she had been told the skin problems were self‐induced. The family were unhappy with this and felt that the skin lesions were not being investigated or treated. During the initial consultation at our clinic, time was spent engaging the family, making them feel heard, and listening to their concerns. The family were given space to air concerns or worries at subsequent appointments. The Consultant Dermatologist provided skin care advice for managing and treating the whole skin and individual skin lesions. This facilitated engagement and once this rapport had been built, an alternative explanation for the skin lesions could be explored. This included a psychiatric assessment by our liaison psychiatry team, who indicated no risk to the young person. The family were provided with a biopsychosocial explanation for skin lesions, in that emotions can be shown on the skin. This enabled exploring how the family managed times of stress and a time line of events including antecedents to the skin lesions, how they were managed by the family (behaviours) and the consequences of this. Little or no emphasis was placed on the mechanism of how the skin lesions appeared. It was highlighted that at times of stress (e.g. hospital admissions, the young person's sister leaving home), the skin lesions would appear. The young person also described being picked on at school which enabled a discussion with school around how to support the young person there. The young person also started to develop non‐epileptic attack disorder and as a result, stopped attending school. This highlighted the importance of actively enquiring about other medically unexplained symptoms. Clinical psychology appointments focussed on stress management and thinking with the family about their reaction to the skin lesions and how they are managed, in order to reduce secondary gains. When these skin lesions appeared, the family were encouraged to treat the skin and then carry on as normal as opposed to the young person being given time off school or doing activities she valued to make her ‘feel better’ (e.g. seeing her sister). After 3 appointments the young person was seen with full resolution of the lesions, with no new lesions being reported from then.

Positive feedback has been received from patients and carers, including:

(i) ‘The sessions with the psychologist are good for *my child* and she receives some useful practical strategies. She is very positive about the sessions’ (F, age 15, Compulsive hair pulling)
(ii) ‘Our expectations were met and more. *The Consultant Dermatologist* went above and beyond with a recommendation to find out what the root cause would be causing *my child's* skin problem. She treated *my child* with so much compassion and care. In fact everyone we came in contact with…. were so lovely. Thank you!’ (F, age 14, Medically unexplained skin signs)
(iii) The family ‘found *the Consultant Dermatologist and the Psychologist* kind, approachable and empathetic. Both were easy to talk to the consultation ended with us feeling hopeful.’ (F, age 14, Trichotillomania)

## 4 DISCUSSION

Our experience supports the growing body of evidence of the effectiveness of a dedicated psychodermatology clinic in managing patients with complex psychocutaneous needs.^5^, ^9^

The consultation model of CYP/psychologist and parent/carer/dermatologist, followed by a joint consultation ensures that both CYP and parent/carers voice are heard within a confidential, non‐judgemental and safe environment. Being able to offer ongoing psychology one to one sessions was key as more than half of our total cohort required these.

A key finding in our analysis was that two children required additional input from CAMHS. This attests to the apparent gap in the provision of services generally for CYP with complex psychodermatology needs, who otherwise do not fulfil criteria to be referred to CAMHS but have important unmet psychological needs. Furthermore CAMHS is at breaking point and in most areas CYP wait many months to be seen. Referrals to CAMHS are often rejected if CYP have skin and hair issues and do not include a significant risk of self‐harm or suicide, as they do not meet the threshold.

Providing a combination of virtual and face‐to‐face appointments improved access to the clinic. For example, there were no *missed* virtual appointments. Verbal feedback revealed that advantages of virtual appointments included reduction in time off from school/homework and work/home commitments for parents. Feedback also revealed that CYP were relaxed and engaged more freely at home being very comfortable interacting within a virtual world. However, there were some limitations to the virtual appointment including building rapport, thorough examination of patient and unpredictable technical drawbacks.

A minority of patients (*n* = 3) had exclusively virtual visits during the Covid‐19 pandemic. We had concerns that this would reduce rapport and effectiveness of the consultation but we had positive feedback from CYP that they felt more comfortable at home and talking to the psychologist whilst in their own bedroom. Post pandemic the majority of patients were offered and accepted a face‐to‐face review at least once, but we found that the combined virtual and face‐to‐face model improved access to psychodermatology multi‐disciplinary team (MDT) care for many more CYP and families who live long distances from the clinic, reduced time off school for CYP and time off work for parents/carers and reduced the number of DNAs in clinic.

Our model of care, characterised by longer appointment times and protected 1:1 work by the CYP and psychologist, and the parent(s) and dermatologist respectively, allows underlying issues including complex family dynamics to be explored in a safe, non‐judgemental space and for collaborative goal setting to occur. Exploration of the CYP/family hopes and expectations on the pre‐clinic questionnaires help guide our consultations.

Our results show the benefit of this model with successful clinical outcomes and discharge from Paediatric dermatology after 1‐2 attendances for many CYP. Taking these complex children out of the general paediatric dermatology clinics reduces pressure on these clinics by reducing stress and anxiety for CYP/parents/carers and the paediatric dermatology clinical team as there is not sufficient time or skill set for a constructive consultation to be had within this setting. Appropriate time for consultations is important not only for comprehensive evaluation of cases but the well being of the clinic staff.^10^

Existing data supports the cost‐effectiveness of *adult* psychodermatology MDT services.^9^ Our discharge data adds to this evidence as 41% of discharged CYP were discharged to primary care with full resolution of their skin problems following a limited number of consultations. This included challenging conditions such as compulsive hair pulling (trichotillomania), medically unexplained skin symptoms (dermatitis artefacta) and skin picking. DeVereHunt et al. reported similar findings within their dedicated dermatology clinic for teenagers with a psychologist available to see all young people.^11^

Part of our consultation is to always explain the mind/skin interaction and to help CYP/parents/carers understand how stress can be expressed by skin and hair signs. We build on this showing how certain behaviours such as compulsive hair pulling and skin picking become coping mechanisms to deal with this in a similar way to nail biting and we aim to destigmatise and de‐shame this behaviour for all. As such some CYP relapse and we have noticed this more since CYP have returned to school post‐lockdown which has been very difficult for many due to academic pressures, exam pressures, peer pressures, increased bullying and the uncertainty of the world today. We therefore have a contact system in place with our Psychologist to allow referral back to the complex clinic if needed. A small number of our patients were referred directly from general practice and we would advocate this as it ensures that the CYP/family are seen in the right setting for all.

Currently, this is one of few dedicated paediatric psychodermatology services nationwide. Our service has excellent feedback from CYP/families and was awarded the Quality in Care Dermatology 2021 prize for Psychodermatology Support Programmes for People with Skin Conditions.^12^ Unfortunately due to Covid a limitation of our data is few fully completed patient reported outcome measures and clinic pre and post questionnaires but we hope now these are linked via clinic codes to DrDoctor (digital patient engagement platform) these should be more complete.

The APPG report (2020) recommend that comprehensive multidisciplinary psychodermatology teams should be commissioned in proportion to patient groups and that access should be at least regional. Current services are few, particularly for CYP and it is vital to provide these at least regionally to prevent long term psychological impact and sequelae for CYP in this difficult world. Our experience indicates that this is an achievable and cost‐effective goal, which can be provided successfully with a hybrid face to face and virtual model and reduces pressure on busy outpatient paediatric general dermatology clinics (Table 1).

**TABLE 1:** Essential features of paediatric psychodermatology/complex clinic

Essential features of paediatric psychodermatology/complex clinic
• Positive working relationships amongst healthcare professionals
• Appropriately timed consultation duration and spacing of consultations
• Face to face consultation at least once during the treatment
• Allow for child and parent voice to be heard separately

## AUTHOR CONTRIBUTIONS

**Alison V. Sears**: Conceptualisation (supporting); Data curation (equal); Formal analysis (equal); Writing – original draft (supporting); Writing – review & editing (equal). **Rukshana Ali**: Conceptualisation (equal); Data curation (lead); Formal analysis (equal); Supervision (equal); Writing – original draft (supporting); Writing – review & editing (equal). **Jane O'Connor**: Data curation (equal); Formal analysis (equal); Writing – original draft (equal). **Susannah Baron**: Conceptualisation (lead); Data curation (equal); Formal analysis (equal); Supervision (lead); Visualisation (lead); Writing – original draft (supporting); Writing – review & editing (lead).

## CONFLICT OF INTEREST

The author declares that there is no conflict of interest that could be perceived as prejudicing the impartiality of the research reported.

## Supporting information

Supporting Information S1

Supporting Information S2

Supporting Information S3

Supporting Information S4

## Data Availability

The data that support the findings of this study are available from the corresponding author upon reasonable request.
