# Supplementary material for: Establishing and developing a paediatric psychodermatology service and our experience of a new paediatric psychodermatology clinic during the Covid 19 pandemic
Source: Skin Health Dis. 2022 Aug 8;2(4):e151. doi: 10.1002/ski2.151 (PMC9539254; doi:10.1002/ski2.151)
Supplement: Supplementary file 2 — Supporting Information S2 [file SKI2-2-e151-s004.pdf]

Appendix 2. Young adult pre-clinic questionnaire

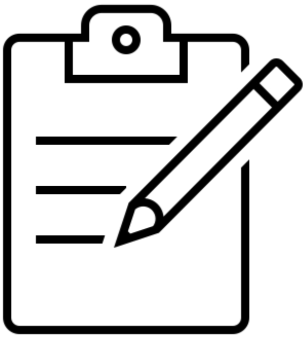

1. How have you been since we last saw you?

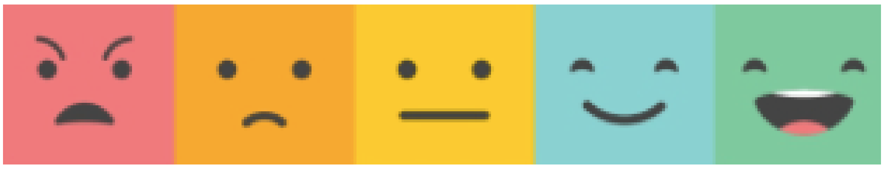

Very bad　　　　　　　　　　　　　　　　　　　　Very good

2. How have these things been in the last month?

	A lot worse	Worse	Same as always	Better	Much Better	N/A
Skin						
Hair						
Scratching						
Picking						
Hair pulling						
Mood						
School						
Friendships						
Home life						

3. What would you like to talk about today? (Tick as many as you like!)

- Mood ☐
- Skin ☐
- Hair ☐
- School ☐
- Friendships ☐
- Home/Family ☐
- Loss of a family member ☐
- Treatment ☐

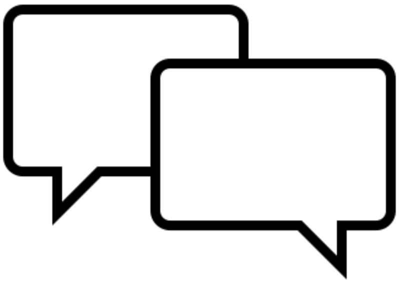

Other: ………………………………………………………

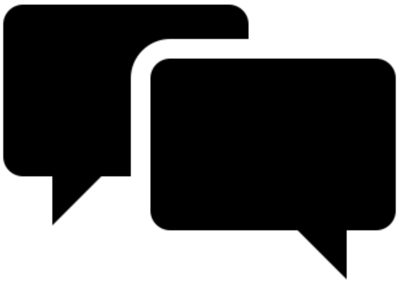

4. Does anyone in the family have?

- Anxiety ☐
- Depression ☐
- Obsessive Compulsive Disorder (OCD) ☐
- Post-Traumatic Stress Disorder (PTSD) ☐

Other: …………………………………………………….

5. What are your hopes for this appointment?

Parent/Caregiver:

Child/Young Person:

6. What would you hope to be different in your life by this time next year?

Parent/Caregiver

Appendix 2. Young adult pre-clinic questionnaire

Child/Young Person

7. Do you use any social media (to be completed by the child/young person)

Snapchat
Instagram
Facebook
Pinterest
Twitter
Other (please state) …………..
N/A
