# Supplementary material for: Establishing and developing a paediatric psychodermatology service and our experience of a new paediatric psychodermatology clinic during the Covid 19 pandemic
Source: Skin Health Dis. 2022 Aug 8;2(4):e151. doi: 10.1002/ski2.151 (PMC9539254; doi:10.1002/ski2.151)
Supplement: Supplementary file 3 — Supporting Information S3 [file SKI2-2-e151-s002.pdf]

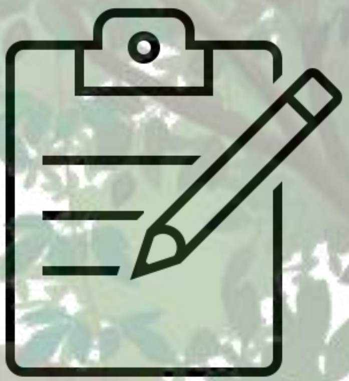

1. **What had you hoped for before the appointment?**

2. **Parent/Caregivers: Did you feel your expectations were me? Please rate this out of 10.**

0	1	2	3	4	5	6	7	8	9	10
Not at all										Completely

3. **Child/Young Person: Did you feel your expectations were met? Please rate this out of 10**

0	1	2	3	4	5	6	7	8	9	10
Not at all										Completely

4. **Parents/Caregivers was it helpful to see the Dermatologist?**
   Yes ☐
   No ☐
   Any additional comments?

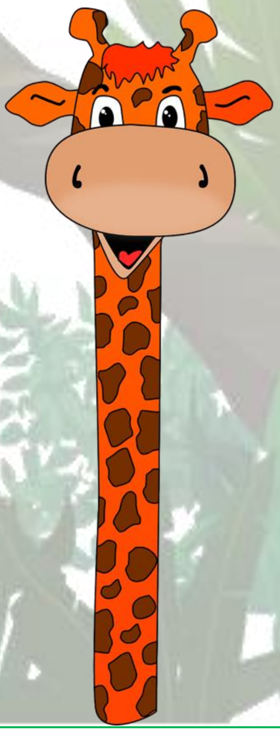

5. **Parents/Caregivers was it helpful to the Psychologist?**
   Yes ☐
   No ☐
   Any additional comments?

6. To the Child/Young: Was it helpful to see the Dermatologist?

Yes ☐

No ☐

Is there anything else you would like to add?

7. To the Child/Young: Was it helpful to see the Psychologist?

Yes ☐

No ☐

Is there anything else you would like to add?

8. What was the most helpful part of the appointment?

9. What could be improved?

10. Is there anything else about the appointment you want to feedback?
