# Supplementary material for: Establishing and developing a paediatric psychodermatology service and our experience of a new paediatric psychodermatology clinic during the Covid 19 pandemic
Source: Skin Health Dis. 2022 Aug 8;2(4):e151. doi: 10.1002/ski2.151 (PMC9539254; doi:10.1002/ski2.151)
Supplement: Supplementary file 4 — Supporting Information S4 [file SKI2-2-e151-s001.pdf]

1. What had you hoped for before the appointment?

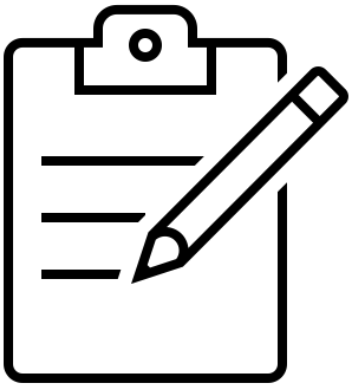

2. Parent/Caregivers: Did you feel your expectations were me? Please rate this out of 10.

0	1	2	3	4	5	6	7	8	9	10
Not at all										Completely

3. Child/Young Person: Did you feel your expectations were met? Please rate this out of 10

0	1	2	3	4	5	6	7	8	9	10
Not at all										Completely

4. Parents/Caregivers was it helpful to see the Dermatologist?
   Yes ☐
   No ☐
   Any additional comments?

5. Parents/Caregivers was it helpful to the Psychologist?
   Yes ☐
   No ☐
   Any additional comments?

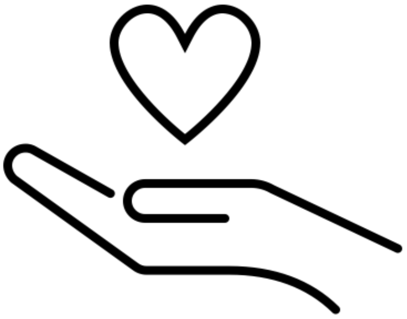

6. To the Child/Young: Was it helpful to see the Dermatologist?

Yes ☐

No ☐

Any additional comments?

7. To the Child/Young: Was it helpful to see the Psychologist?

Yes ☐

No ☐

Any additional comments?

8. What was the most helpful part of the appointment?

9. What could be improved?

10. Is there anything else about the appointment you want to feedback?
